# Sierra Leone: the forgotten mortality

**DOI:** 10.3402/gha.v8.26757

**Published:** 2015-02-09

**Authors:** Vageesh Jain, Colin Stewart Brown, Oliver Johnson

**Affiliations:** 1 King’s College London, London, UK; 2 King’s Centre for Global Health, King’s Health Partners, King’s College London, London, UK

The 2014 Ebola virus disease (EVD) outbreak is the largest in history, affecting multiple countries across West Africa, Europe, and the United States. Sierra Leone is among the predominant three nations affected, alongside Guinea and Liberia. As of 14 December 2014, there have been 8,356 cases of Ebola in Sierra Leone, with 2,417 deaths (1). However, little focus has been paid to the deterioration of healthcare in these already fragile West African states. 

Though 15% of gross domestic product was previously being spent on healthcare (2), with a gross national income of only $680 per capita in 2013 (3), health was already stretched in a country with striking disease statistics. For example, tuberculosis (TB) contributes dramatically to the burden of disease in Sierra Leone, which has the third-highest prevalence of TB in the world (4). Though the number of incident infections of HIV has been declining, Sierra Leone has 57,000 people living with HIV. Access to antiretroviral (ARV) drugs had been steadily increasing over the past decade, though the total percentage with access was under 50% of those recommended for treatment initiation (5). Since the EVD crisis, TB and HIV delivery systems have been curtailed, with fragmentation of long-term care. In both cases, drug resistance, increased community transmission, and unnecessary death are all to be expected. Sierra Leone ranks fifth in the world for number of malaria cases, with approximately 85% of the population with access to insecticide-treated mosquito nets, but just under 20% with access to indoor residual spraying (6).

Sierra Leone also has one of the world's highest rates of maternal mortality, ranking fifth in the world (7). One in 10 women risks dying during pregnancy or childbirth, a rate relatively unchanged since 1990 (8). Sierra Leone ranks first in the world for under-five mortality, with almost one in every three children dying before reaching the age of five (9).

Despite progress in some areas of healthcare over the past few years, Sierra Leone faces the same challenges as it did more than a decade ago. EVD has decimated the provision of healthcare within the country. Obstructed labour, existent infectious disease threats, paediatric vaccination programmes, chronic non-communicable disease care all still remain but much is going untreated. We must not lose sight of these indicators as we continue to combat the spread of Ebola. We must look to strengthen the existing health system as well as deliver urgent interventions to stop EVD spread. We must consider how EVD treatment can bolster healthcare, and not solely build parallel systems that fail to complement each other.

King's Sierra Leone Partnership is working closely with our government partners to keep Connaught Hospital, the major referral hospital in the country, open and functioning. The international community must do more to address the dual challenges of EVD and the fragmentation and loss of all existing healthcare, if Sierra Leone is to have any future ability to cope with the health demands of its population. Despite previous objections to any health systems strengthening being discussed, by the end of December 214, UN Mission for Ebola Emergency Response (UNMEER) recognised that global EVD response in itself was not enough, that past efforts have neglected rebuilding of health systems, and that ‘Ebola has destroyed whatever hopes these three countries might have had for even moderate prosperity and human security’ (10). National health system strengthening must therefore be a core tenant to the future EVD response, along with long-term investment once the last case is seen. Though this is far from being realised, let us hope that the ‘forgotten mortality’ of EVD in Sierra Leone does not remain the legacy of EVD in West Africa.

*Vageesh Jain* King's College London London, UK Email: vageesh.jain@kcl.ac.uk *Colin Stewart Brown* King's Centre for Global Health, King's Health Partners King's College London London, UK *Oliver Johnson* King's Centre for Global Health, King's Health Partners King's College London London, UK

## References

[1] World Health Organization Ebola response roadmap situation report update. http://apps.who.int/iris/bitstream/10665/144117/1/roadmapsitrep_21Nov2014_eng.pdf.

[2] WHO Sierra Leone. http://www.who.int/countries/sle/en/.

[3] The World Bank Data – Sierra Leone. http://data.worldbank.org/country/sierra-leone.

[4] Public Health England (2012). WHO estimates of Tuberculosis incidence by country. https://www.gov.uk/government/uploads/system/uploads/attachment_data/file/332777/TB_Worldwide_by_Country_2012.pdf.

[5] UNAIDS 2014 country progress reports. http://www.unaids.org/en/dataanalysis/knowyourresponse/countryprogressreports/2014countries/SLE_narrative_report_2014.pdf.

[6] WHO World malaria report 2013. http://www.afro.who.int/en/clusters-a-programmes/dpc/malaria/features/3998-world-malaria-report-2013.html.

[7] The World Factbook Country comparison: maternal mortality rate. https://www.cia.gov/library/publications/the-world-factbook/rankorder/2223rank.html.

[8] United Nations Population Fund The state of the world's midwifery. http://www.unfpa.org/sowmy.

[9] World Health Organization WHO country cooperation strategy. http://www.who.int/countryfocus/cooperation_strategy/ccs_sle_en.pdf.

[10] Horton R (2014). Offline: can Ebola be a route to nation-building?. Lancet.

